# AMPK–mTORC1 pathway mediates hepatic IGFBP-1 phosphorylation in glucose deprivation: a potential molecular mechanism of hypoglycemia-induced impaired fetal growth

**DOI:** 10.1530/JME-23-0137

**Published:** 2024-01-31

**Authors:** Jenica H Kakadia, Muhammad U Khalid, Ilka U Heinemann, Victor K Han

**Affiliations:** 1 Department of Biochemistry, Schulich School of Medicine and Dentistry, The University of Western Ontario, London, Ontario, Canada; 2 Children's Health Research Institute, London, Ontario, Canada; 3 Department of Pediatrics, Schulich School of Medicine and Dentistry, The University of Western Ontario, London, Ontario, Canada

**Keywords:** maternal nutrient restriction, insulin-like growth factor-1, TOR serine-threonine kinases, glucose deprivation, fetal hypoglycemia, fetal growth restriction, fetal liver, fetal hepatocytes

## Abstract

Mechanisms underlying limitations in glucose supply that restrict fetal growth are not well established. IGF-1 is an important regulator of fetal growth and IGF-1 bioavailability is markedly inhibited by IGFBP-1 especially when the binding protein is hyperphosphorylated. We hypothesized that the AMPK–mTORC1 pathway increases IGFBP-1 phosphorylation in response to glucose deprivation. Glucose deprivation in HepG2 cells activated AMPK and TSC2, inhibited mTORC1 and increased IGFBP-1 secretion and site-specific phosphorylation. Glucose deprivation also decreased IGF-1 bioavailability and IGF-dependent activation of IGF-1R. AICAR (an AMPK activator) activated TSC2, inhibited mTORC1, and increased IGFBP-1 secretion/phosphorylation. Further, siRNA silencing of either AMPK or TSC2 prevented mTORC1 inhibition and IGFBP-1 secretion and phosphorylation in glucose deprivation. Our data suggest that the increase in IGFBP-1 phosphorylation in response to glucose deprivation is mediated by the activation of AMPK/TSC2 and inhibition of mTORC1, providing a possible mechanistic link between glucose deprivation and restricted fetal growth.

## Introduction

Glucose is the primary energy source for fetal metabolism and growth. Glucose is transported across the placenta via facilitated diffusion mediated by glucose transporters, in particular, human glucose transporter 1 (GLUT1) (Jansson *et al.* 1999, Acosta *et al.* 2015). Fetal growth restriction (FGR) is defined as a failure of the fetus to reach its genetic growth potential, a condition affecting 3–7% of all infants (Shrivastava & Master 2020). FGR increases risk of perinatal and neonatal complications, and predisposes the infant for developing diabetes and cardiovascular disease in childhood and adult life (Barker *et al.* 1989, Limesand *et al.* 2019). Although the causes of FGR are multifactorial, reduced oxygen and nutrient transport from the mother to the fetus due to placental insufficiency is believed to be the most common cause in the developed world (Wollmann 1998).

Although the precise pathophysiological mechanisms underlying FGR have not been fully established, studies have implicated the insulin-like growth factor (IGF) system to play an important role (Holmes *et al.* 1997). IGFs are critical for optimal fetal and placental growth and development, secreted by the fetal liver and placenta in pregnancy (Martina *et al.* 1997) and act in endocrine, paracrine, and autocrine mechanisms to regulate cellular growth, differentiation and apoptosis (Forbes & Westwood 2008). IGF-1 is an important regulator of fetal growth and IGF-1 action is modulated by IGF binding proteins (IGFBP) 1-6 (Clemmons 1989). IGFBP-1, secreted by the fetal liver, is most abundant in fetal life and is believed to be the most important fetal IGFBP. When IGFBP-1 is phosphorylated, the affinity for binding IGF-1 increases markedly, thereby further decreasing the bioavailability of the growth factor to stimulate cellular growth, differentiation and apoptosis (Baxter 2000, Abu Shehab *et al.* 2010). Thus, phosphorylation of IGFBP-1 is an important additional mechanism regulating IGF-1 action in fetal growth (Gupta 2015). We have shown previously that phosphorylation of fetal IGFBP-1 is increased in human FGR and in a baboon maternal nutrient restriction model of FGR (Abu Shehab *et al.* 2010, Gupta 2015, Kakadia *et al.* 2020, 2021), and may directly contribute to the decreased fetal growth in this pregnancy complication. A better understanding of the molecular mechanisms regulating IGFBP-1 phosphorylation will provide a new insight into the pathophysiology of FGR and lead to potential interventions.

FGR fetuses are often hypoglycemic *in utero* (Economides *et al.* 1989, Economides & Nicolaides 1989), which may contribute to FGR development. Decreased levels of glucose and amino acids in the umbilical vein may be associated with perinatal brain injury leading to neurological impairment in FGR infants (Malhotra *et al.* 2017). In healthy pregnancy, fetal gluconeogenesis is largely inactive and fetal glucose needs are met by transplacental transport of glucose. However, during prolonged periods of glucose starvation, gluconeogenesis is induced in fetal hepatocytes (Joyner *et al.* 2016). Indeed, increased fetal liver expression of gluconeogenic enzymes has been reported in several animal models of FGR, including in rats fed a low-protein diet (Liu *et al.* 2013) and in baboons with maternal nutrient restriction (Nijland *et al.* 2010). Thus, fetal liver glucose homeostasis adapts to decreased nutrient delivery in FGR fetuses.

We have reported previously that hypoxia and amino acid deprivation induce fetal hepatic IGFBP-1 hyperphosphorylation through the mechanistic target of rapamycin (mTOR) and amino acid response pathways, respectively (Seferovic *et al.* 2009, Kakadia *et al.* 2021). However, the mechanisms by which glucose deprivation alters IGFBP-1 phosphorylation is currently unknown. We hypothesized that AMPK (5′adenosine monophosphate-activated protein kinase), a key energy sensor, is activated by glucose deprivation (Lyons & Roche 2018) which then stimulates IGFBP-1 secretion and increases phosphorylation via decreased mTORC1 activity. AMPK is an obligate, heterotrimeric, 63 kDa metabolic regulator upstream of mTOR (Xiao *et al.* 2011). The catalytic α-subunit of AMPK is phosphorylated at Thr172 by the serine–threonine liver kinase B1, which is activated by increasing AMP:ATP ratio (Xiao *et al.* 2011). Following Thr172 phosphorylation, AMPK phosphorylates and activates an array of downstream effectors to promote ATP-generating catabolic processes (Mihaylova & Shaw 2011). One of the targets of AMPK is the tumor suppressor tuberous sclerosis complex 2 (TSC2), directly upstream of mTORC1 pathway. When TSC2 is phosphorylated by AMPK at Ser1387, mTORC1 is inhibited, resulting in several downstream effects, including increased autophagy and decreased cell growth and proliferation (Mihaylova & Shaw 2011).

mTOR is a serine–threonine protein kinase that forms the catalytic subunit of the two complexes mTORC1 and mTORC2 (Yang *et al.* 2013). mTORC1 promotes protein synthesis via the phosphorylation of p70S6 kinase 1 (P70S6K1) and eIF4E binding protein (4E-BP1). mTORC2, on the other hand, plays roles in survival and proliferation by phosphorylating proteins like Akt (Saxton & Sabatini 2017). AMPK does not directly affect mTORC2 signaling. Instead, AMPK prevents the induction of cell proliferation and protein synthesis by inhibiting mTORC1, thus resulting in decreased cell growth. We have reported previously that IGFBP-1 is hyperphosphorylated in response to hypoxia and is mediated by the inhibition of mTORC1 (Damerill *et al.* 2016). However, it is unknown if AMPK-mediated mTORC1 inhibition links glucose starvation to IGFBP-1 phosphorylation. AMPK may inhibit mTORC1 through different phosphorylation events. One potential mechanism is the phosphorylation and activation of TSC2, and downstream inhibition of mTORC1 (Lyons & Roche 2018); AMPK may also phosphorylate and inhibit a component of mTORC1, the regulatory-associated protein of mTOR (Raptor) at Ser792 (Gwinn *et al.* 2008, Hardie 2008).

In this study, using pharmacological and siRNA approaches in hepatocellular carcinoma HepG2 cells (as a model of fetal hepatocytes) cultured with and without glucose, we tested the hypothesis that the AMPK–mTORC1 pathway increases IGFBP-1 phosphorylation in response to glucose deprivation. We showed that in glucose deprivation, liver AMPK and TSC2 signaling is activated, and subsequent mTORC1 inhibition may mediate IGFBP-1 hyperphosphorylation.

## Methods

### Cell culture and treatments

For the *in vitro* studies, we used human hepatocellular carcinoma HepG2 (ATCC-HB-8065) cells, which have extensive similarity to primary human fetal hepatocytes (Kelly & Darlington 1989, Wilkening *et al.* 2003). HepG2 cells were cultured in Dulbecco’s modified Eagle medium (DMEM)–F-12 (Cat # 11320033, Thermo Fisher Scientific), supplemented with 10% fetal bovine serum (Cat #10082147, FBS, Thermo Fisher Scientific). Cells were grown in atmospheric air (20% O_2_ with 5% CO_2_) at 37°C. Cells were then plated in six-well plates at 400,000 cells/well and allowed to attach for 16–24 h. Cells were subsequently exposed to pharmacological treatments, siRNA and/or glucose deprivation. Following treatment, cells were placed on ice and cell media were collected. Cells were washed with Dulbecco’s phosphate-buffered saline (Cat #D8537, DPBS, Sigma-Aldrich) and 150 μL lysis buffer (1:1000 protease inhibitor cocktail (Cat #P8340, Sigma-Aldrich), 1:1000 phosphatase inhibitor cocktail 2 (Cat #P5726, Sigma-Aldrich), 1:1000 phosphatase inhibitor cocktail 3 (Cat #P0044, Sigma-Aldrich)) with 10× cell lysis buffer (Cat #9803, Cell Signaling Technologies) in water were additionally added to each well. Cells were frozen at −80°C overnight, and subsequently scraped, sonicated for 45 s, and centrifuged for 30 min at 10,000 ***g***. The cell lysate was stored at −20°C until analyses.

### Glucose deprivation treatment

α-d(+)-Glucose (Cat #G8270, Sigma-Aldrich) was added to DMEM (no glucose) media (Cat #11966025, Thermo Fisher Scientific) to a final concentration of 17.5 mM for use as control media; 0 mM glucose DMEM media was used as the glucose deprivation media. Glucose concentrations of 0 to 17.5 mM were used to study the effects of glucose deprivation on HepG2 cells, and based on the concentration-dependent (Supplementary Fig. 1, see the section on supplementary materials given at the end of this article) effect on IGFBP-1 secretion and phosphorylation, 0 mM of glucose was chosen to study the effect of glucose deprivation. Cell viability was not changed at 36 h after glucose deprivation compared to controls (Supplementary Fig. 2). Ten percent FBS was added to the media throughout the growth of the cells, with the exception of starvation, when cells were starved with 2% FBS DMEM media. Following plating of cells, starvation media was added to each well and cells were starved overnight. Media was then replaced with either control or glucose deprivation media for 36 h, after which cell media and lysate were collected.

### Pharmacological treatment

AICAR (5-aminoimidazole-4-carboxamide-1-β-d-ribofuranoside) is an activator of AMPK, acting as an analog of adenosine monophosphate (AMP). AICAR is cell-permeable and can bind to AMPK subunits with high affinity, leading to AMPK activation (Hardie 2008). AMPK activator A-769662, also known as A76, was also used; this molecule mimics the effects of AMP by allosteric activation and inhibiting dephosphorylation (Göransson *et al.* 2007, Huang *et al.* 2020, Kim *et al.* 2022). HepG2 cells were plated and starved as described previously. For AICAR treatment, cells were treated with 1 mM DMSO (Cat#DMS666, BioShop Canada Inc., ON, Canada) as the control treatment, or 1 mM AICAR (Cat #ab120358, Abcam) to induce AMPK activity. For A76 treatment, cells were treated with 100 μM DMSO (Cat#DMS666, BioShop Canada Inc., ON, Canada) as the control treatment, or 100 μM A76 (Cat# ab120335, Abcam) to induce AMPK activity. Cells were treated for 36 h in DMEM–F-12 media and subsequently cell media and cell lysate were collected.

### RNA interference-mediated silencing

HepG2 cell signaling was targeted with specific siRNA silencing of AMPK (30 nM; Sigma-Aldrich) or TSC2 (100 nM; Sigma-Aldrich) using Dharmafect transfection reagent 4 (Cat #T-2004, Thermo Fisher Scientific). HepG2 cells were then treated with control scramble siRNA (Cat #SIC001, Sigma-Aldrich) or specific siRNA targeting AMPK or TSC2 in 10% FBS DMEM–F-12 media for 24 h. Cell media was subsequently exchanged for 10% FBS DMEM–F-12 media for 24 h and then cells were serum-starved with 0% FBS DMEM–F-12 media for 6 h. Cells were treated with control or glucose deprivation media as described earlier. Both cell media and lysate were collected for analyses, and the efficiency of target silencing was determined at the protein level using western blot analysis.

### Cell proliferation assay

Cells were treated with glucose deprivation with or without siRNA targeting AMPK or TSC2. Using a cell proliferation kit (Cat # 11465007001, MTT, Sigma-Aldrich), 24 h after the addition of media with or without glucose, 200 μL of MTT solution was added to each well. Cells were incubated for 4 h at 37°C. Two milliliters MTT solubilization buffer were then added to each well, and cells were rocked to ensure adequate distribution. Cells were incubated overnight at 37°C. The cell media with buffer was thoroughly mixed to assure solubilization of the colorimetric marker, and absorbance was read at 595 nm. A negative control, with cell media and buffer only, no cells, was read alongside for normalization.

### IGF-1 receptor assay

P6 cells (derived from mouse fibroblast BALB/c3T3 cells) overexpressing human IGF-1R (obtained from Dr Renato Baserga, Thomas Jefferson University, PA, USA), were plated at 380,000 cells/well in 6-well plates in 10% FBS high-glucose DMEM with sodium pyruvate and geneticin. Geneticin was used as a selective agent for P6 cells overexpressing human IGF-1R. P6 cells do not secrete IGFBP-1. This model was used as a functional assay to determine IGF-1 bioactivity, by assessing IGF-1R autophosphorylation as previously described (Malkani *et al.* 2015, Nandi *et al.* 2021). P6 cells were serum-starved with 0% FBS high-glucose DMEM with sodium pyruvate for 6 h prior to treatment. Conditioned HepG2 media was collected from glucose deprivation treatments, and total IGFBP-1 was measured using normalization to stock samples with known IGFBP-1 concentrations, determined by ELISA as described previously (Malkani *et al.* 2015, Nandi *et al.* 2021). This conditioned media containing known amounts of IGFBP-1 was incubated for 2 h at room temperature with human recombinant IGF-1 in a 2 ng IGF-1–200 ng IGFBP-1 ratio, to allow the formation of IGFBP-1–IGF-1 complexes. Phosphorylated IGFBP-1, which has an increased binding affinity for IGF-1, should bind more IGF-1. Following starvation, P6 cells were treated for 10 min as follows: with IGF-1 (100 ng/mL) in the absence of IGFBP-1 as a positive control; no IGF-1 as a negative control; or the previously mentioned conditioned media containing IGF-1–IGFBP-1 complexes.

This experiment controlled the total amount of IGFBP-1, as conditioned media used to treat P6 cells would maximally contain 200 ng IGFBP-1 per well, but phosphorylation of IGFBP-1 differed between treatments. Thus, this allowed us to observe the effect of IGFBP-1 phosphorylation on IGF-1 bioavailability as shown by IGF-1Rβ autophosphorylation at Tyr1135. Following treatment of P6 cells overexpressing human IGF-1R, cell media was aspirated, cells were rinsed with PBS and 150 μL of cell lysis buffer was added to the wells. Cell lysates were prepared as described earlier and IGF-1Rβ phosphorylation at Tyr 1135 was analyzed via western blotting.

### SDS-PAGE and Western blotting

Cell lysate was quantified and equal amounts of total protein (50–100 μg) was used to determine phosphorylation and total expression of Raptor (Cat# 2280) at Ser792 (Cat# 2083), TSC2 (Cat# 4308) at Ser1387 (Cat# 5584), AMPK (Cat# 2532) at Thr172 (Cat# 2535), p70S6K1 (Cat# 9202) at Thr389 (Cat# 9205), 4E-BP1 (Cat# 9644) at Thr70 (Cat# 9455), Akt (Cat# 9272) at Ser473 (Cat# 9271), pACC at Ser79 (Cat#3661), ACC (Cat#3662), and IGF-1Rβ (Cat# sc-390130, Santa Cruz Biotechnology) at Tyr1135 (Cat# 3918); antibodies were obtained from Cell Signaling Technology. To confirm equal loading, β-actin (Cat# 3700, Cell Signaling Technology) was used as a loading control during quantification. The IGFBP-1 polyclonal antibody was a gift from Dr Robert Baxter (Kolling Institute of Medical Research, Sydney, Australia). Custom IGFBP-1 polyclonal antibodies targeting Ser101, Ser119, and Ser169 were generated at YenZym Antibodies LLC (Brisbane, CA, USA), and have been previously validated (Abu Shehab *et al.* 2013, 2014). Equal volume loading of cell media was performed to determine IGFBP-1 secretion and phosphorylation at Ser101, Ser119, and Ser169.

Blots were blocked with 5% skim milk in Tris-buffered saline plus 0.1% Tween 20 (TBS-T; Cat #TWN510, BioShop) for 1 h at room temperature. Primary antibodies were used at a dilution of 1:1,000 except for total IGFBP-1 antibody at 1:10,000, pIGFBP-1^Ser101^ at 1:500, pIGFBP-1^Ser169^ at 1:250, and β-actin at 1:3,000. Cells were probed with primary antibodies overnight at 4°C. Peroxidase-labeled anti-mouse (Cat# 1706516, Bio-Rad Laboratories) or anti-rabbit (Cat# 1706515, Bio-Rad Laboratories) antibodies (1:10,000) were used as secondary antibodies. Secondary antibody was applied for 1 h at room temperature and blots were subsequently visualized by in-house enhanced chemiluminescence (ECL) reagents and imaged on a VersaDoc MP Imager (Bio-Rad).

### Statistical analysis

Western blots band intensities were quantified with densitometry using Image Lab software (Bio-Rad), and statistical analyses were performed using GraphPad Prism 5 (GraphPad Software). Treatment groups were normalized to controls and controls were assigned an arbitrary value of 1 to facilitate comparisons. Protein of interest amounts were normalized to loading controls prior to statistical analyses. Unpaired *t*-test and one-way ANOVA were used to compare means, specified in figure legends; results were expressed as mean + s.d., with *P* < 0.05 considered significant.

## Results

### Glucose deprivation increases IGFBP-1 phosphorylation

**Figure 1:**
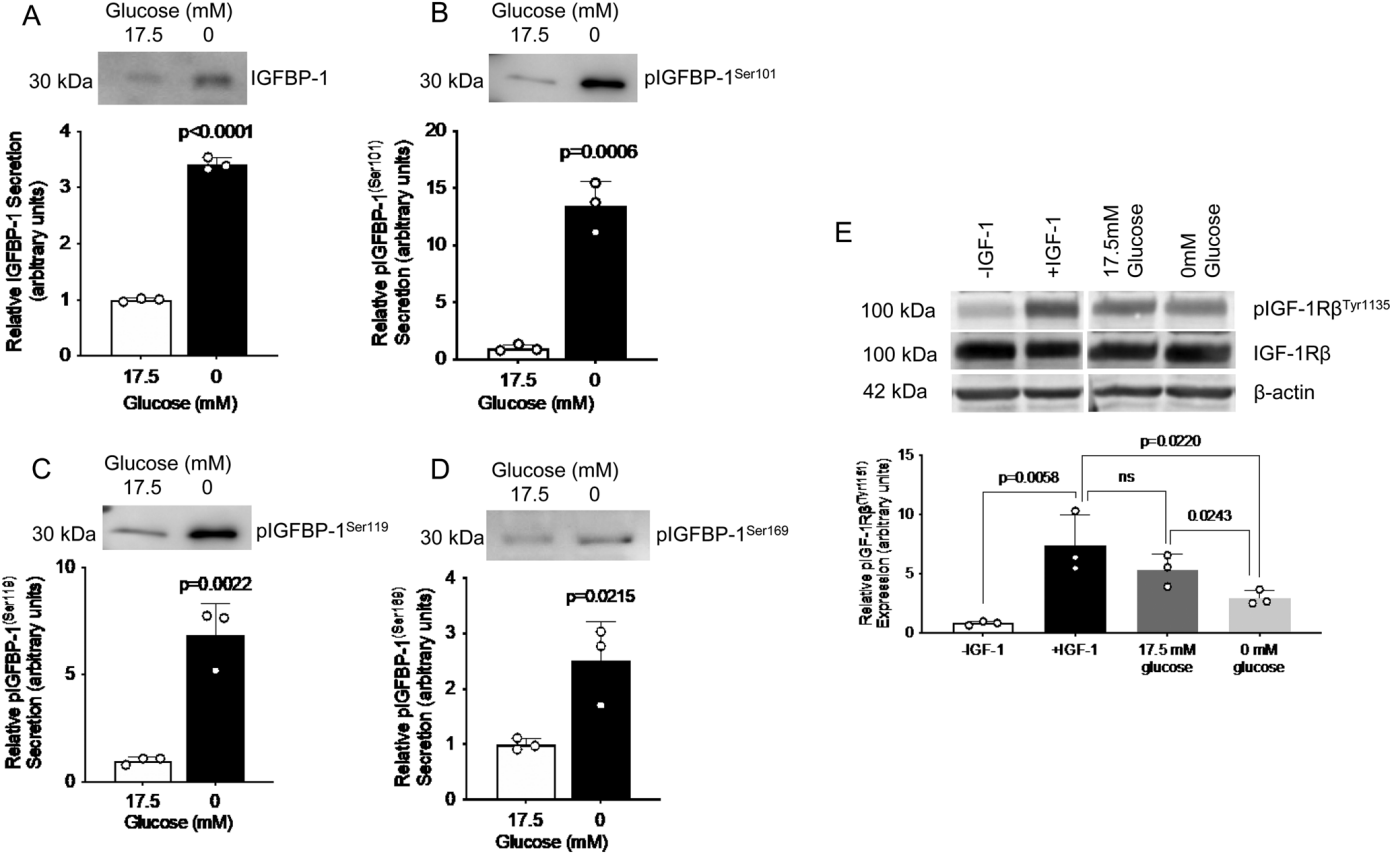
Glucose deprivation increases IGFBP-1 secretion and phosphorylation in HepG2 cells and decreases IGF-1Rβ autophosphorylation in P6 cells. Representative western blots of total IGFBP-1 abundance (A) and IGFBP-1 phosphorylation at Ser101 (B), Ser119 (C), and Ser169 (D) in HepG2 cell media following a 36-h treatment with glucose deprivation. Equal aliquots of cell media were loaded (10 μL to detect total IGFBP-1; 30 μL to detect phospho-IGFBP-1). Bar graph shows means + s.d., with treated groups normalized against controls and controls were arbitrarily assigned an average of 1. Unpaired *t-*test, *n* = 3 all, *P* < 0.05 is considered significant. A representative western blot of IGF-1R autophosphorylation at Tyr1135 and total IGF-1R in P6 cells (E). Equal concentrations of total IGFBP-1 from conditioned HepG2 cell media with or without glucose deprivation were used to induce IGF-1R autophosphorylation in P6 cells overexpressing human IGF-1R. Equal loading of P6 cell lysate was performed (50 μg) to detect IGF-1Rβ (Tyr1135) autophosphorylation. Negative and positive controls with or without IGF-1, respectively, were used for normalization. One-way ANOVA with Bonferroni correction for multiple comparisons test, *n* = 3, *P* < 0.05 is considered significant.

We have previously shown that nutrient deficiency, including amino acid (leucine) deprivation and hypoxia, increase IGFBP-1 secretion and phosphorylation in HepG2 cells (Seferovic *et al.* 2009, Malkani *et al.* 2015, 2016, Damerill *et al.* 2016). However, whether glucose deprivation similarly induces IGFBP-1 phosphorylation is not known. In this study, we first examined the effects of glucose deprivation on IGFBP-1 secretion and phosphorylation in HepG2 cell media. HepG2 cells were cultured in DMEM with either 0 mM (glucose deprivation) or 17.5 mM (control) glucose concentrations for 36 h. Standard DMEM/F-12 media used to culture HepG2 cells contains 17.5 mM glucose; hence, we used this concentration of glucose for an appropriate control. As well, a dose-dependency experiment using varying concentrations of glucose from 0 to 17.5 mM (Supplementary Fig. 1A, B, and C) showed 0 mM of glucose significantly induced of IGFBP-1 compared to control. Although complete glucose deprivation may not be seen in the fetus, in this study, we utilize a complete deprivation of glucose as a pharmacologic stimulation to investigate the mechanisms that regulate IGFBP-1 phosphorylation in glucose deprivation, as the concentration of 0 mM glucose offers a significant increase in IGFBP-1 secretion similar to that observed in fetal growth restriction *in vivo*. Indeed, partial glucose deprivation can lead to FGR, impair organ development, and alter metabolic programming, increasing the risk of metabolic disorders such as obesity and type 2 diabetes in adulthood (Thorn *et al.* 2011, Devaskar & Chu 2016), and can also affect placental function and nutrient transfer (Fowden *et al.* 2006). We demonstrated that glucose deprivation induced a significant increase in the secretion of total IGFBP-1 (3.4-fold increase, *P* < 0.001), pIGFBP-1^Ser101^ (13.5-fold increase, *P* < 0.001), pIGFBP-1^Ser119^ (6.9-fold increase, *P* = 0.002) and pIGFBP-1^Ser169^ (2.5-fold increase, *P* = 0.022) relative to controls, as seen by western blotting (Fig. 1A, B, C, and D). Thus, IGFBP-1 secretion and phosphorylation were strongly induced in glucose deprivation at 36 h in HepG2 cells.

### Glucose deprivation reduces IGF-1 bioavailability due to increased IGFBP-1 phosphorylation

To determine the effects of glucose deprivation on IGF-1 bioavailability, an IGF-1R autophosphorylation assay was performed using P6 cells, which were engineered to overexpress human IGF-1R. P6 cells were either incubated without IGF-1 (-IGF-1, negative control), IGF-1 alone (+IGF-1, positive control) or HepG2 cell media containing equal total IGFBP-1, complexed with IGF-1, from cells treated with (17.5 mM) glucose or without (0 mM) glucose. P6 cells showed a 7.4-fold increase in IGF-1R autophosphorylation following the addition of IGF-1, compared to the negative control (Fig. 1E, *P* = 0.006), supporting that bioavailable IGF-1 is capable of inducing IGF-1R autophosphorylation in P6 cells. HepG2 cells secrete basal levels of IGFBP-1 (Damerill *et al.* 2016) which sequesters some IGF-1, as seen when P6 cells were incubated with control HepG2 cell media + IGF-1 compared to IGF-1 alone. However, the change was not significant. Importantly, incubation of P6 cells with conditioned media from HepG2 cells treated with glucose deprivation significantly reduced IGF-1R autophosphorylation compared to the positive control (0.4-fold decrease, *P* = 0.022). As glucose deprivation increased relative IGFBP-1 phosphorylation compared to control glucose conditions, the phosphorylation events should further increase the affinity of IGFBP-1 for IGF-1. This was seen as P6 cells treated with glucose deprivation media had a significant reduction in IGF-1R autophosphorylation (0.5-fold decrease, *P* = 0.024) compared to cells treated with control glucose media, containing equal total IGFBP-1 levels (lanes 3 and 4). Since the total IGFBP-1 was kept at equal concentrations, this data suggests that decreased IGF-1R autophosphorylation was due to decreased IGF-1 bioavailability mediated by increased IGFBP-1 phosphorylation, rather than overall IGFBP-1 levels.

### Glucose deprivation activates AMPK and TSC2 and inhibits mTORC1 activity

**Figure 2:**
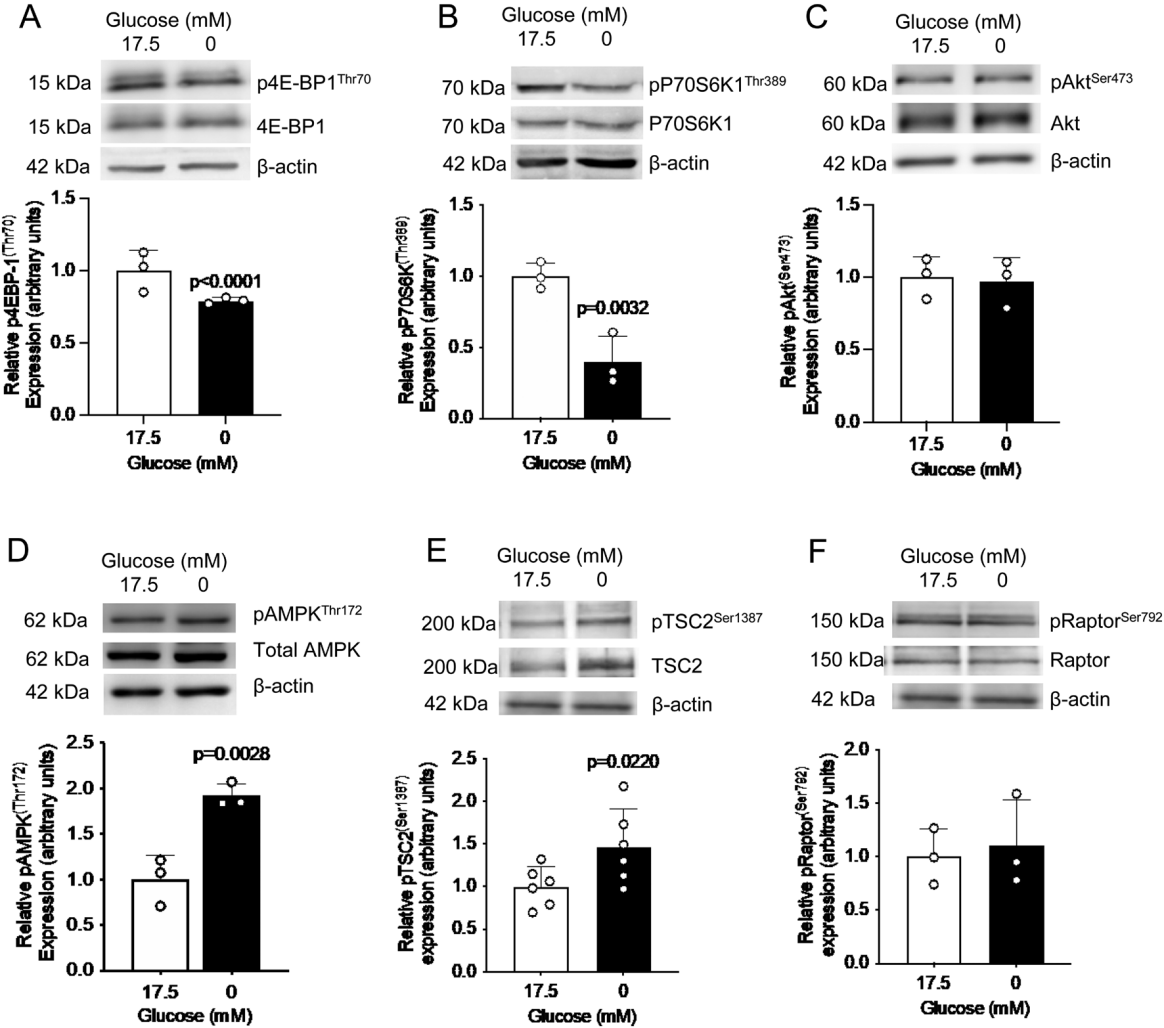
Glucose deprivation inhibits mTORC1 activity and activates AMPK and increases TSC2 phosphorylation in HepG2 cells. Representative western blots of p4E-BP1^Thr70^ (A), pP70S6K1^Thr389^ (B), and pAkt^Ser473^ (C), pAMPK^Thr172^ (D), pTSC2^Ser1387^ (E), and pRaptor^Ser792^ (F) in HepG2 cell lysate following treatment with glucose deprivation. Equal loading of lysate was performed (50 μg). Bar graph shows means + s.d., with treated groups normalized against controls and controls were arbitrarily assigned an average of 1. Unpaired *t-*test, *n* = 3 (A, B, C, D, F) and *n* = 6 (E), *P* < 0.05 is considered significant.

To elucidate whether the AMPK–mTORC1 pathway mediates IGFBP-1 phosphorylation in glucose-deprived HepG2 cells, we first determined whether AMPK activation and mTORC1 inhibition occurred in glucose deprivation. We found mTORC1 is inhibited as seen by decreased phosphorylation of its functional readouts, p4E-BP1^Thr70^ (0.8-fold decrease, *P* < 0.001, Fig. 2A) and pP70S6K1^Thr389^ (0.5-fold decrease, *P* = 0.003, Fig. 2B). However, mTORC2 activity is unchanged, as seen by no change in the phosphorylation of its functional readout, pAkt^Ser473^ (Fig. 2C). Upstream, we determined that pAMPK^Thr172^ was increased (1.9-fold increase, *P* = 0.003, Fig. 2D) and therefore might be responsible for mTORC1 inhibition in glucose deprivation. Phosphorylation of AMPK at Thr172 is the mechanism for its activation; its activity is stimulated more than 100-fold by phosphorylation at this site (Willows *et al.* 2017).

AMPK has been shown to phosphorylate two key proteins in order to inhibit mTORC1: TSC2 at Ser1387 and Raptor at Ser792 (Gwinn *et al.* 2008, Van Nostrand *et al.* 2020). To determine whether only one or both phosphorylation events are mediating mTORC1 inhibition in glucose deprivation, we determined the phosphorylation of TSC2 and Raptor using western blotting. We showed that only pTSC2^Ser1387^ was increased in glucose deprivation (1.4-fold increase, *P* = 0.022), while pRaptor^Ser792^ was unchanged (Fig. 2E and F). Together, this data suggests the AMPK–mTORC1 pathway, via TSC2 phosphorylation and activation, likely plays an important role in glucose deprivation signaling.

### AICAR activates AMPK and TSC2 and inhibits mTORC1 activity

**Figure 3:**
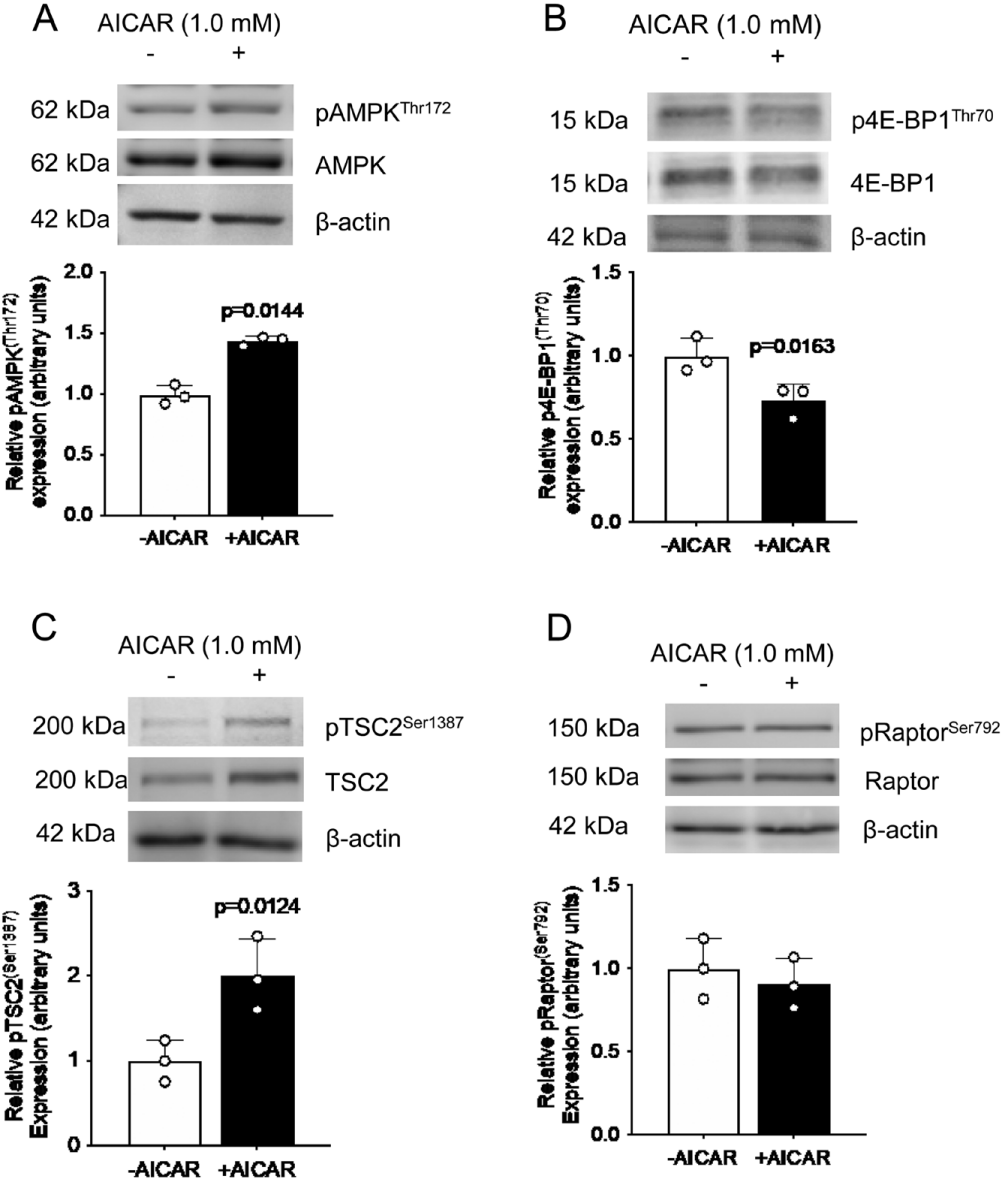
AMPK activation by AICAR inhibits mTORC1 activity and increases TSC2 phosphorylation in HepG2 cells. Representative western blots of pAMPK^Thr172^ (A), p4E-BP1^Thr70^ (B), pTSC2^Ser1387^ (C), and pRaptor^Ser792^ (D) in HepG2 cell lysate following treatment with the AMPK activator AICAR in normal glucose conditions. Equal loading of lysate was performed (50 μg). Bar graph shows means + s.d., with treated groups normalized against controls and controls were arbitrarily assigned an average of 1. Unpaired *t-*test, *n* = 3 all, *P* < 0.05 is considered significant.

AICAR is a potent activator of AMPK. To determine whether the changes in mTORC1 inhibition and TSC2 activation in glucose deprivation were due to AMPK, we utilized this AMPK activator in control glucose conditions. After treating HepG2 cells with AICAR in control glucose conditions, we confirmed that AMPK was activated (1.4-fold increase, *P* = 0.014, Fig. 3A), and showed that AMPK activation inhibited mTORC1 activity as seen by decreased phosphorylation of its functional readout p4E-BP1^Thr70^ (0.8-fold decrease, *P* = 0.016, Fig. 3B).

Additionally, we determined if AMPK activation mediates TSC2 and/or Raptor phosphorylation. We found that AICAR induced pTSC2^Ser1387^ (1.7-fold increase, *P* = 0.028), while pRaptor^Ser792^ was unchanged (Fig. 3C andD). In addition to TSC2 phosphorylation, its expression was also increased. This result showed that AMPK activation might be responsible for the effects seen in glucose deprivation, where mTORC1 was inhibited and TSC2 phosphorylation was increased at Ser1387.

### AICAR increases IGFBP-1 phosphorylation

**Figure 4:**
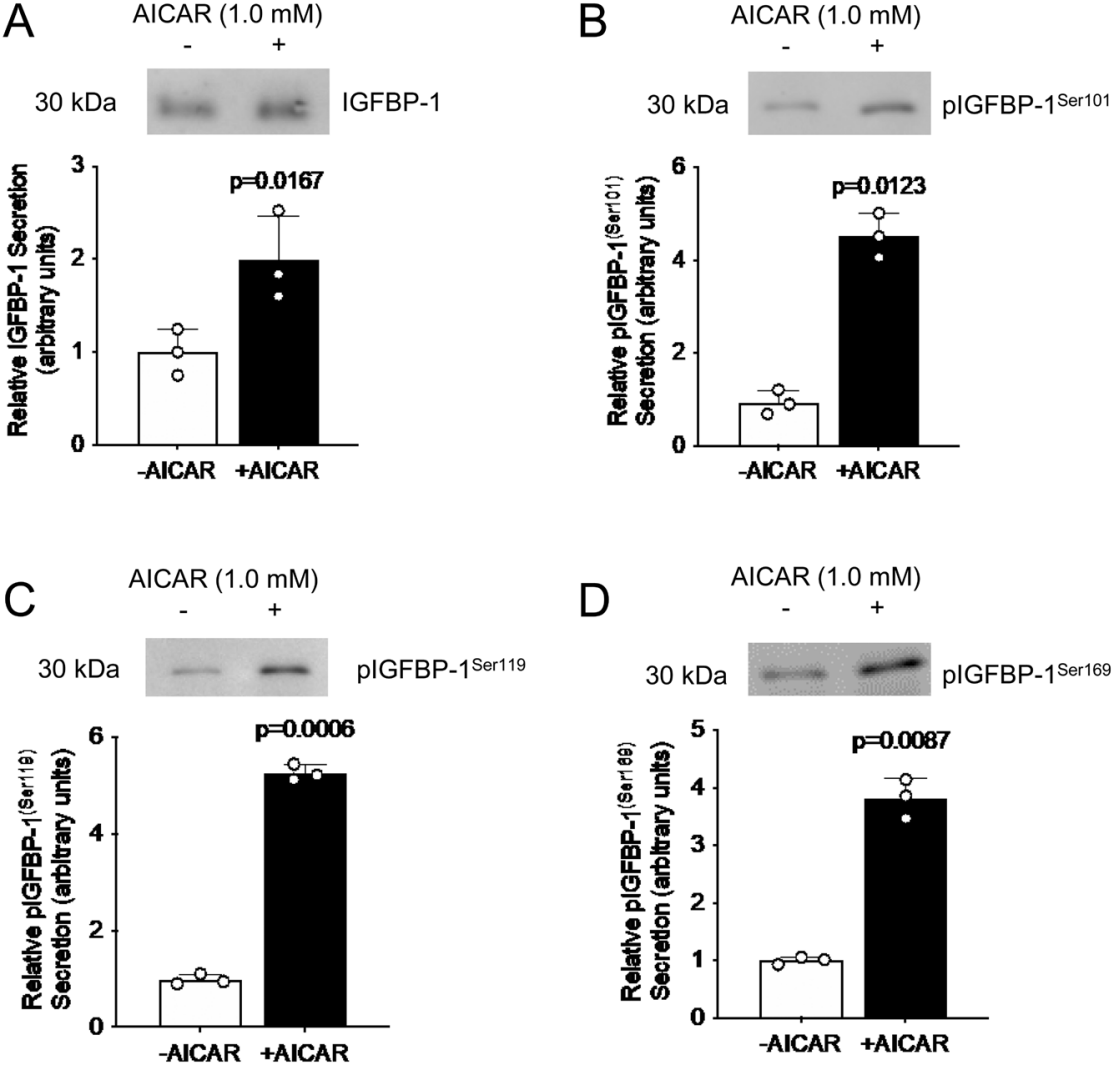
AMPK activation by AICAR increases IGFBP-1 secretion and phosphorylation in HepG2 cells. Representative western blots of total IGFBP-1 abundance (A) and IGFBP-1 phosphorylation at Ser101 (B), Ser119 (C), and Ser169 (D) in HepG2 cell media following treatment with AICAR, an AMPK activator. Equal aliquots of cell media were loaded (10 μL for total IGFBP-1; 30 μL for pIGFBP-1). Bar graph shows means + s.d., with treated groups normalized against controls and controls were arbitrarily assigned an average of 1. Unpaired *t-*test, *n* = 3 all, *P* < 0.05 is considered significant.

To further investigate whether AMPK signaling mediates increased IGFBP-1 secretion and phosphorylation, we utilized AICAR as a pharmacologic activator of AMPK. We determined the effects on IGFBP-1 secretion and phosphorylation in HepG2 cell media following AICAR treatment using western blotting. We showed that AICAR induced a significant increase in the secretion of total IGFBP-1 (2.0-fold increase, *P* = 0.017), pIGFBP-1^Ser101^ (4.6-fold increase, *P* = 0.012), pIGFBP-1^Ser119^ (5.3-fold increase, *P* < 0.001), and pIGFBP-1^Ser169^ (3.7-fold increase, *P* = 0.009) relative to controls (Fig. 4A, B, C and D). Thus, AMPK activation by AICAR is responsible for IGFBP-1 secretion and phosphorylation.

### A76 activates AMPK signaling and TSC2 phosphorylation

**Figure 5:**
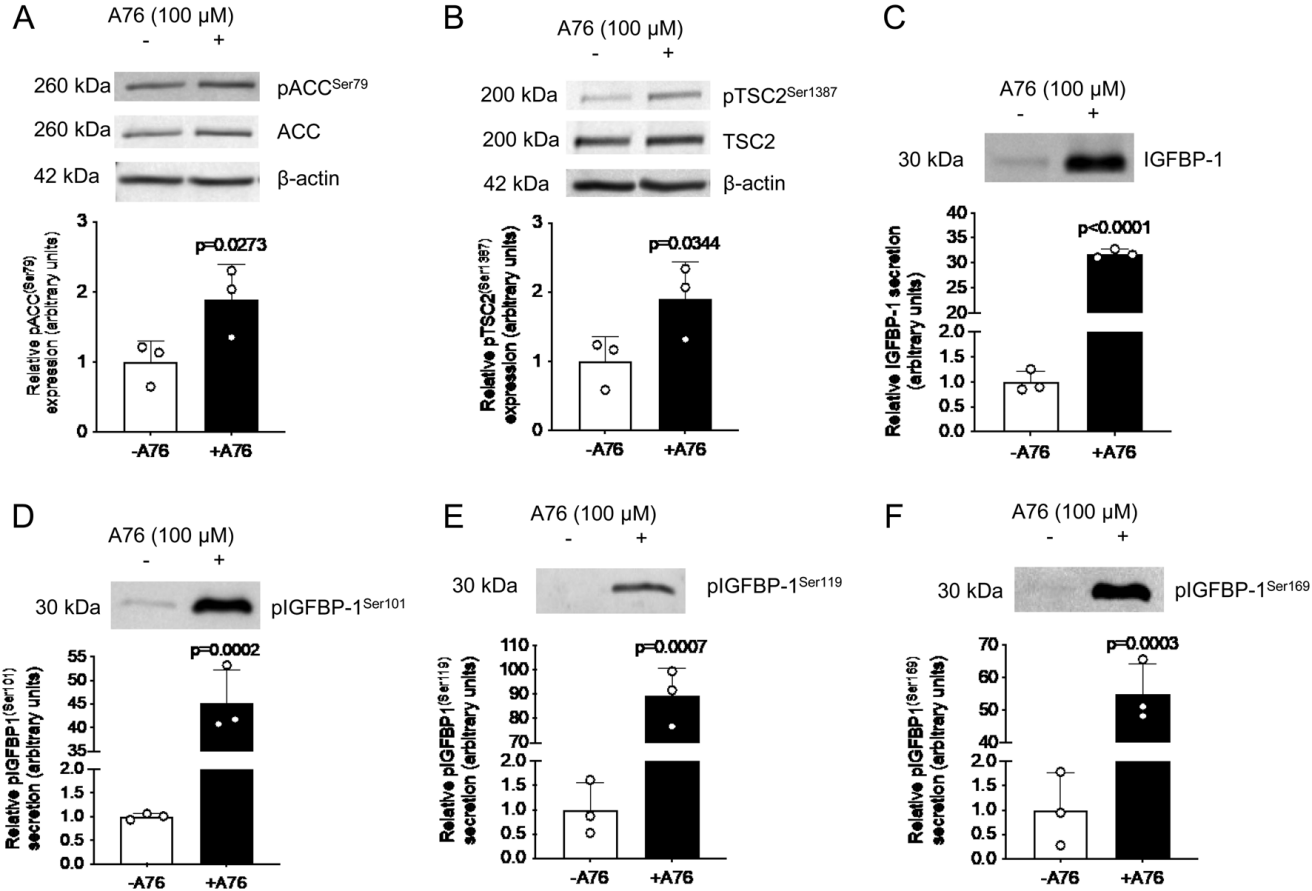
AMPK activation by A76 increases TSC2 phosphorylation and IGFBP-1 secretion and phosphorylation in HepG2 cells. Representative western blots of pACC^Ser79^ (A) and pTSC2^Ser1387^ (B) in HepG2 cell lysate following treatment with the AMPK activator A76 in normal glucose conditions. Equal loading of lysate was performed (50 μg). Representative western blots of total IGFBP-1 abundance (C) and IGFBP-1 phosphorylation at Ser101 (D), Ser119 (E), and Ser169 (F) in HepG2 cell media following treatment with A76. Equal aliquots of cell media were loaded (30 μL for total IGFBP-1; 40 μL for pIGFBP-1). Bar graph shows means + s.d., with treated groups normalized against controls and controls were arbitrarily assigned an average of 1. Unpaired *t-*test, *n* = 3 all, *P* < 0.05 is considered significant.

To further confirm the involvement of AMPK as pharmacological activators may have off-target effects, we additionally treated cells with another AMPK activator, A-769662, also known as A76. A76 mimics the effects of AMP by allosteric activation and inhibiting dephosphorylation (Göransson *et al.* 2007, Huang *et al.* 2020, Kim *et al.* 2022). We utilized this AMPK activator in control glucose conditions, and confirmed that AMPK activity was activated, as seen by the increased phosphorylation of the downstream substrate acetyl COA carboxylase (ACC) at Ser 79 (1.9-fold increase, *P* = 0.027, Fig. 5A). We also found that this AMPK activation increased pTSC2^Ser1387^ (1.9-fold increase, *P* = 0.034) (Fig. 5B). Thus, AMPK activation may be responsible for the effects seen in glucose deprivation.

### A76 increases IGFBP-1 phosphorylation

To further determine whether AMPK signaling mediates IGFBP-1 phosphorylation, we treated HepG2 cells with A76, a pharmacological activator of AMPK. We showed that A76 induced a significant increase in the secretion of total IGFBP-1 (31-fold increase, *P* < 0.001), pIGFBP-1^Ser101^ (45-fold increase, *P* < 0.001), pIGFBP-1^Ser119^ (89-fold increase, *P* < 0.001) and pIGFBP-1^Ser169^ (55-fold increase, *P* < 0.001) relative to controls (Fig. 5C, D, E, and F). Thus, AMPK activation by A76 increases IGFBP-1 secretion and phosphorylation.

### AMPK siRNA silencing prevents glucose-deprivation mediated TSC2 activation and mTORC1 inhibition

**Figure 6:**
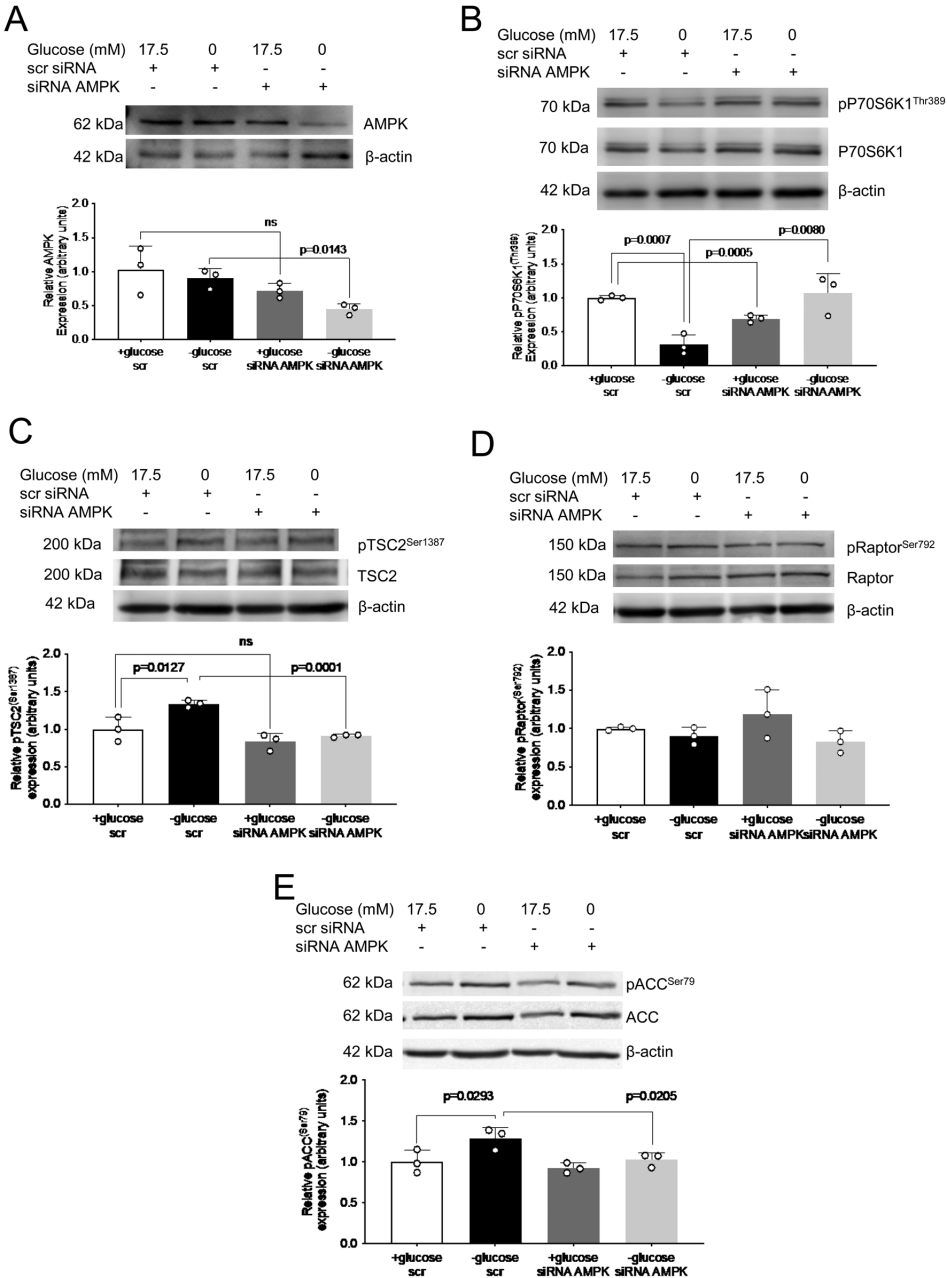
AMPK siRNA effectively silences AMPK and prevents mTORC1 inhibition and TSC2 phosphorylation in glucose deprived HepG2 cells. Representative western blots of AMPK (A), pP70S6K1^Thr389^ (B), pTSC2^Ser1387^ (C), pRaptor^Ser792^ (D), and pACC^Ser79^ in HepG2 cell lysate following treatment with glucose deprivation and/or AMPK siRNA silencing. Equal loading of lysate was performed (50 μg). Bar graph shows means + s.d., with treated groups normalized against controls and controls were arbitrarily assigned an average of 1. One-way ANOVA with Bonferroni correction for multiple comparisons test, *n* = 3 all, *P* < 0.05 is considered significant.

To confirm the mechanistic involvement of AMPK in mediating IGFBP-1 phosphorylation in glucose deprivation, we silenced AMPK using targeted siRNA in HepG2 cells with or without glucose deprivation. Although AMPK siRNA did not have significant effects on AMPK expression in control conditions, significant silencing efficiency of AMPK was seen in glucose deprived conditions (0.5-fold decrease, *P* = 0.014, Fig. 6A). While glucose deprivation inhibited mTORC1 (0.3-fold decrease, *P* < 0.001), AMPK siRNA in glucose deprivation prevented mTORC1 inhibition, as seen by pP70S6K1^Thr389^ (3.4-fold increase, *P* = 0.008, Fig. 6B).

Similarly, increased TSC2 phosphorylation at Ser1387 in glucose deprivation (1.3-fold increase, *P* = 0.013) was prevented when AMPK was silenced (0.7-fold decrease, *P* < 0.001, Fig. 6C); TSC2 phosphorylation was unaffected in control conditions by AMPK siRNA treatment. Raptor phosphorylation at Ser792 remained unchanged (Fig. 6D). Additionally, to confirm these effects are a result of AMPK activity, we probed for pACC^Ser79^, a downstream target of AMPK which is increased during AMPK activation, and found that the increase in pACC^Ser79^ in glucose deprivation (1.3-fold increase, *P* = 0.029) is prevented by AMPK silencing (0.2-fold decrease, *P* = 0.021) (Fig. 6E). These data show that AMPK mediates increased TSC2 phosphorylation and mTORC1 activation in glucose deprivation.

### AMPK siRNA silencing prevents glucose-deprivation mediated IGFBP-1 phosphorylation

**Figure 7:**
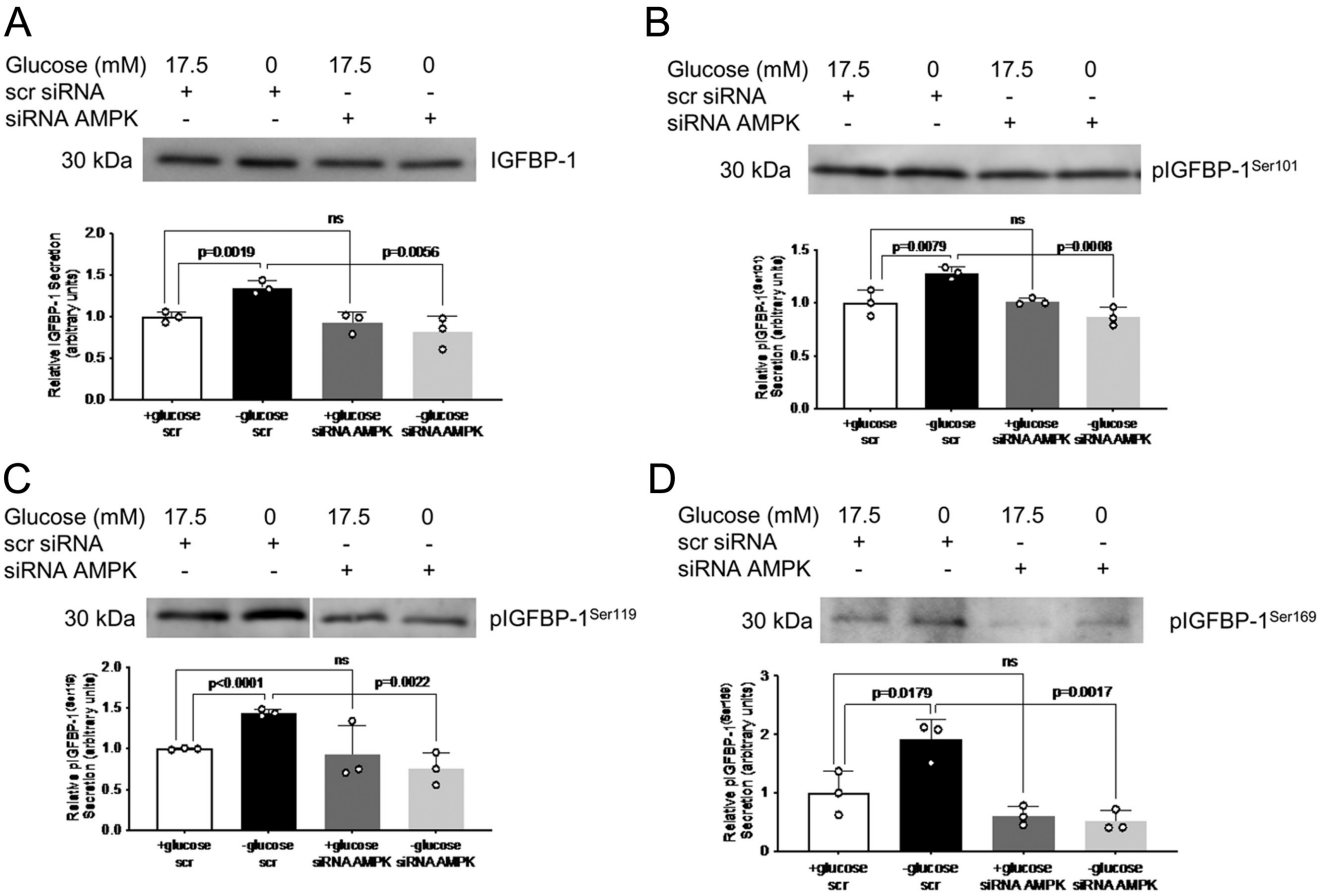
AMPK silencing by siRNA prevents IGFBP-1 secretion and phosphorylation in glucose deprived HepG2 cells. Representative western blots of total IGFBP-1 abundance (A) and IGFBP-1 phosphorylation at Ser101 (B), Ser119 (C), and Ser169 (D) in HepG2 cell media following treatment with glucose deprivation and/or siRNA targeting AMPK. Equal aliquots of cell media were loaded (10 μL for total IGFBP-1; 30 μL for pIGFBP-1). Bar graph shows means + s.d., with treated groups normalized against controls and controls were arbitrarily assigned an average of 1. One-way ANOVA with Bonferroni correction for multiple comparisons test, *n* = 3 all, *P* < 0.05 is considered significant.

We used AMPK siRNA-treated HepG2 cells in control or glucose deprivation conditions to determine whether IGFBP-1 is still hyperphosphorylated under glucose deprivation when AMPK is silenced. Although IGFBP-1 secretion (1.4-fold increase, *P* = 0.002) and phosphorylation at Ser101 (1.3-fold increase, *P* = 0.008), Ser119 (1.4-fold increase, *P* < 0.001), and Ser169 (1.9-fold increase, *P* = 0.018) were increased by glucose deprivation, inhibition of AMPK expression by siRNA in glucose deprivation prevented this increased secretion (0.6-fold decrease, *P* = 0.006) and phosphorylation of IGFBP-1 at Ser101 (0.7-fold decrease, *P* < 0.001), Ser119 (0.5-fold decrease, *P* = 0.002), and Ser169 (0.3-fold decrease, *P* = 0.002) (Fig. 7A, B, C, and D). Neither IGFBP-1 secretion nor phosphorylation was affected by AMPK silencing in control conditions. This, together with the previous data using siRNA-inhibited AMPK expression in control or glucose deprivation conditions, confirms the involvement of the AMPK–TSC2–mTORC1 pathway in regulating IGFBP-1 secretion and phosphorylation in glucose deprivation.

### TSC2 siRNA silencing prevents glucose-deprivation mediated mTORC1 inhibition and IGFBP-1 phosphorylation

**Figure 8:**
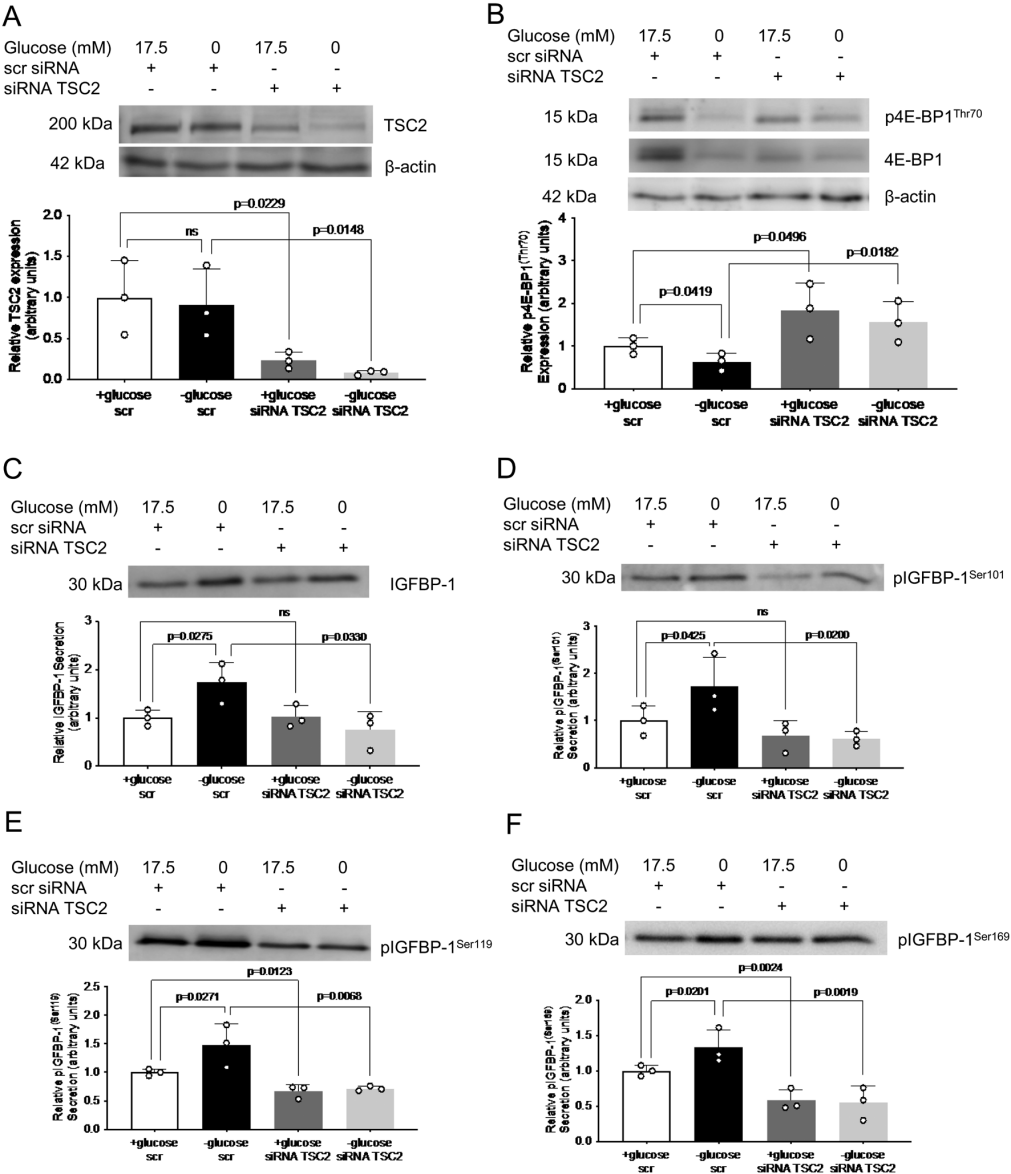
TSC2 siRNA effectively silences TSC2 and prevents mTORC1 inhibition and IGFBP-1 secretion and phosphorylation. In glucose deprived HepG2 cells. Representative western blot of TSC2 (A) and p4E-BP1^Thr70^ (B) in HepG2 cell lysate following treatment with glucose deprivation and/or siRNA targeting TSC2. Equal loading of lysate was performed (50 μg). Representative western blots of total IGFBP1 abundance (C) and IGFBP-1 phosphorylation at Ser101 (D), Ser119 (E), and Ser169 (F) in HepG2 cell media following treatment with glucose deprivation and/or siRNA targeting TSC2. Equal aliquots of cell media were loaded (10 μL for total IGFBP-1; 30 μL for pIGFBP-1). Bar graph shows means +s.d., with treated groups normalized against controls and controls were arbitrarily assigned an average of 1. One-way ANOVA with Bonferroni correction for multiple comparisons test, *n* = 3 all, *P* < 0.05 is considered significant.

Next, to determine whether TSC2 activation is necessary in mediating the effects of glucose deprivation on mTORC1 inhibition and increased IGFBP-1 phosphorylation, we silenced TSC2 expression with specific siRNA. As expected, no difference was seen in TSC2 expression in control vs glucose deprivation conditions (Fig. 8A). Appropriate silencing efficiency of TSC2 was seen in control conditions (0.8-fold decrease, *P* = 0.023) and glucose deprivation (0.1-fold decrease, *P* = 0.015, Fig. 8A). While glucose deprivation inhibited mTORC1 signaling (0.5-fold decrease, *P* = 0.042), TSC2 silencing in glucose deprivation prevented mTORC1 inhibition as seen by p4E-BP1^Thr70^ (2.5-fold increase, *P* = 0.018) (Fig. 8B). TSC2 siRNA also increased p4E-BP1^Thr70^ (1.8-fold increase, *P* = 0.049) in control conditions (Fig. 8B).

When determining changes in IGFBP-1 secretion/phosphorylation in the same conditions, we found that while glucose deprivation alone increased IGFBP-1 secretion (1.6-fold increase, *P* = 0.028) and phosphorylation at Ser101 (1.7-fold increase, *P* = 0.043), Ser119 (1.4-fold increase, *P* = 0.027), and Ser169 (1.3-fold increase, *P* = 0.020), TSC2 silencing in glucose deprivation prevented increased IGFBP-1 secretion (0.6-fold decrease, *P* = 0.033) and phosphorylation at Ser101 (0.3-fold decrease, *P* = 0.020), Ser119 (0.6-fold decrease, *P* = 0.007), and Ser169 (0.5-fold decrease, *P* = 0.002) (Fig. 8C, D, E, and F). Neither IGFBP-1 secretion nor phosphorylation at Ser101 was affected by TSC2 silencing in control conditions; however, TSC2 silencing decreased IGFBP-1 phosphorylation at Ser119 (0.7-fold decrease, *P* = 0.012) and Ser169 (0.6-fold decrease, *P* = 0.002), suggesting complex downstream TSC2 signaling on multiple kinases to control different phosphorylation events in control conditions (Fig. 8C, D, E, and F). These data provide evidence that TSC2 signaling is an important regulator of mTORC1 inhibition and IGFBP-1 secretion and phosphorylation in glucose deprivation.

### Glucose deprivation decreases cell proliferation

**Figure 9:**
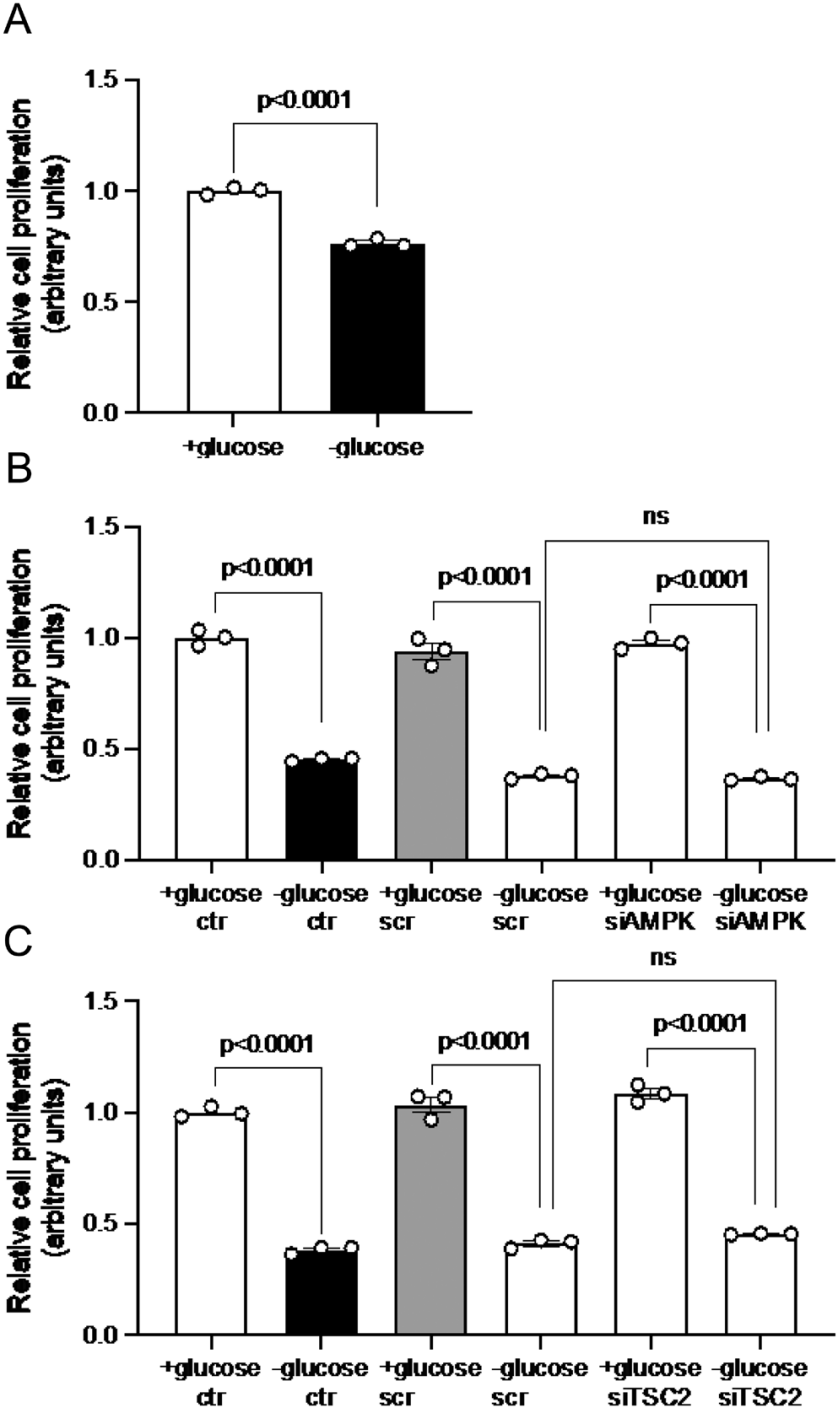
Cell proliferation assay of HepG2 cells. HepG2 cells were cultured with or without glucose (A), as well as with or without AMPK siRNA (B) or TSC2 siRNA (C) as described previously. An MTT assay was used to determine cell proliferation and absorbance was plotted in bar graphs showing mean + s.d. Absorbance was normalized against a negative control and controls were arbitrarily assigned an average of 1. Unpaired *t-*test, or one-way ANOVA with Bonferroni correction for multiple comparisons test, *n* = 3, *P* < 0.05 is considered significant.

TSC2 and mTORC1 are key regulators of cell growth and metabolism (Saxton & Sabatini 2017). To determine whether changes in signaling during glucose deprivation affect cell proliferation, we utilized an MTT cell proliferation assay for cells treated with glucose deprivation, with or without AMPK or TSC2 siRNA as described earlier. The absorbance measured of the colorimetric marker represents relative cell proliferation. We found that glucose deprivation alone significantly reduced cell proliferation (0.8-fold decrease, *P* < 0.001, Fig. 9A). Furthermore, glucose deprivation decreased cell proliferation compared to controls in control conditions (0.4-fold decrease, *P* < 0.001), as well as after the addition of scrambled siRNA (0.4-fold decrease, *P* < 0.001) or siRNA targeting AMPK (0.4-fold decrease, *P* < 0.001); silencing of AMPK did not affect cell proliferation compared to scrambled control in glucose deprivation (Fig. 9B). Similarly, glucose deprivation decreased cell proliferation compared to controls in control conditions (0.4-fold decrease, *P* < 0.001) as did the addition of scrambled siRNA (0.4-fold decrease, *P* < 0.001) or siRNA targeting TSC2 (0.4-fold decrease, *P* < 0.001, Fig. 9C). TSC2 silencing also did not affect cell proliferation compared to scrambled siRNA in glucose deprivation (Fig. 9C).

## Discussion

Using gene silencing and pharmacologic approaches in HepG2 cells, this study showed that activation of AMPK and TSC2 and the resulting inhibition of mTORC1 mediates IGFBP-1 phosphorylation in response to glucose deprivation. Increased IGFBP-1 phosphorylation likely inhibits the actions of IGF-1 (Yu *et al.* 1998, Abu Shehab *et al.* 2013), an important regulator of fetal growth (Bauer *et al.* 1998, Martín-Estal *et al.* 2016). Additionally, because human FGR fetuses are often hypoglycemic (Economides *et al.* 1989, Economides & Nicolaides 1989, Adamkin 2017), our data provides new insights into the pathophysiology of FGR. We recognize that our study using an *in vitro* cell model cannot definitively establish mechanisms; however, our findings provide insight into the potential mechanisms at play, which can guide future *in vivo* studies and provide a basis for further investigation.

Together with insulin (Economides *et al.* 1989, Economides & Nicolaides 1989), IGF-1 is believed to be the most important growth stimulating endocrine and paracrine factor in fetal development. IGFBP-1 limits IGF-1 bioavailability by sequestering IGF-1, thereby inhibiting the growth-promoting actions of IGF-1 (Clemmons 1989); IGFBP-1 binding affinity for IGF-1 is further increased when it is phosphorylated at key serine residues (Ser101, Ser119, Ser169) (Abu Shehab *et al.* 2013). Increased IGFBP-1 levels are implicated in reduced fetal growth (Ben Lagha *et al.* 2006) and we have previously reported increased IGFBP-1 secretion and hyperphosphorylation in human FGR (Abu Shehab *et al.* 2010, 2014, Gupta *et al.* 2019). In this study, we showed that IGF-1R activation is reduced in glucose deprivation by the increased levels of phosphorylated IGFBP-1, supporting that IGFBP-1 phosphorylation plays an important role in mediating IGF-1 bioavailability under this growth restricting condition. Human FGR fetuses are often hypoglycemic (Adamkin 2017); however, mechanistic links between decreased glucose availability and increased fetal liver IGFBP-1 secretion and phosphorylation have not been established.

In this study, glucose deprivation in HepG2 cells markedly increased secretion and phosphorylation of IGFBP-1. Consistent with previous reports (Lyons & Roche 2018), AMPK was significantly activated and mTORC1 was inhibited by glucose deprivation. mTORC1 is a regulator of protein synthesis, mitochondrial function, nutrient transport and autophagy in response to signaling by nutrients, oxygen and growth factors, thereby modulating cell proliferation and growth (Jhanwar-Uniyal *et al.* 2019). In this study, we showed that glucose deprivation significantly decreases cellular proliferation. Withdrawal of glucose, amino acids or oxygen leads to rapid suppression of mTORC1 activity (Shaw & Cantley 2006). In agreement with these known regulators of mTOR signaling, we demonstrated that mTORC1 was inhibited by glucose deprivation, resulting in increased IGFBP-1 secretion and phosphorylation while mTORC2 remained unaffected.

AMPK is activated in response to various conditions and signals that indicate decreased cellular energy, including nutrient deprivation or prolonged starvation (Hardie 2007, Lin & Hardie 2018) due to the decreased ATP availability. Animal models have shown the physiological importance of hepatic AMPK for whole-body glucose homeostasis (Viollet *et al.* 2006, Hardie *et al.* 2012); AMPK plays a major role in the control of hepatic metabolism. In HepG2 cells, we have previously reported that liver mTORC1 is inhibited and IGFBP-1 phosphorylation is increased during hypoxia and amino acid deprivation (Seferovic *et al.* 2009, Malkani *et al.* 2015, Damerill *et al.* 2016). These observations suggest that activation of AMPK in the fetal liver may represent a key event linking nutrient deprivation to the inhibition of mTORC1 and increased IGFBP-1 secretion and phosphorylation.

AMPK activation can inhibit mTORC1 activity (Krause *et al.* 2002, Reiter *et al.* 2005) through multiple mechanisms (Inoki *et al.* 2012), including phosphorylation and activation of TSC2 at Ser1387, a negative regulator of mTORC1 (Leprivier & Rotblat 2020), and/or phosphorylation of Raptor, a component of mTORC1, resulting in inhibition of mTORC1 (Gwinn *et al.* 2008). mTORC1 inhibition following activation of AMPK causes slowing of cell growth and increased autophagy in response to energy deprivation (Mihaylova & Shaw 2011). In this study, we showed that AMPK activation results in the phosphorylation of TSC2 but not Raptor, suggesting a preferential pathway for mTORC1 inhibition during glucose deprivation. Additionally, the loss of TSC2 activity by siRNA silencing resulted in activation of mTORC1 but not mTORC2, confirming that TSC2 is a negative regulator of mTORC1 in HepG2 cells. TSC2 activation via its phosphorylation constitutes one key mechanism by which glucose and oxygen control mTORC1 activity (Inoki *et al.* 2003). Our data suggests that following activation in response to glucose deprivation, AMPK directly phosphorylates TSC2 on conserved serine sites. TSC2 phosphorylation, therefore, represents a specific mechanism by which mTORC1 senses and responds to glucose availability. A robust increase in IGFBP-1 phosphorylation in the absence of glucose (0 mM) may not be physiological. HepG2 cells were exposed to complete glucose deprivation for 36 h, whereas the FGR fetuses *in vivo* may be subjected to hypoglycemia for a prolonged period of weeks to months. Indeed, poor nutrient transfer to the fetus, such as due to placental insufficiency, results in chronic hypoglycemia, causing metabolic changes to preserve glucose and induces hepatic gluconeogenesis (Yates *et al.* 2011). Additionally, we recognize that conducting cell experiments in atmospheric oxygen (21%) does not reflect the fetal oxygen levels. These experiments were done in normoxia as HepG2 cells are adapted to and thrive in a normoxic environment. Lower oxygen levels (e.g. 10% or lower, as seen in fetal development) in conjunction with glucose deprivation could increase cell stress, and confound results. Future experiments will show if these findings are translatable to FGR reveal if chronic hypoglycemia elicits IGFBP-1 hyperphosphorylation *in vivo* similar to the degree observed in the *in vitro* studies in this report.

**Figure 10:**
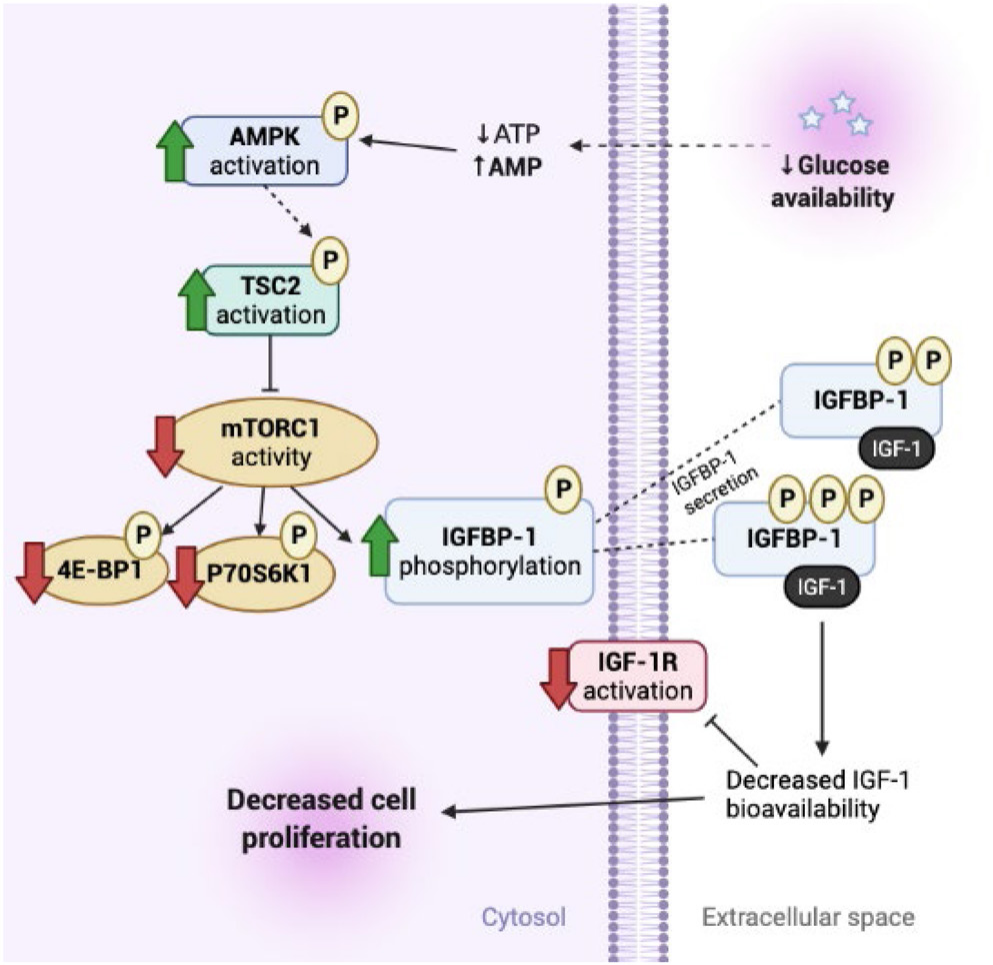
Proposed model of the molecular mechanisms linking glucose deprivation to increased IGFBP-1 secretion and phosphorylation. AMPK inhibits mTORC1 activity via phosphorylation of TSC2; mTORC1 inhibition increases IGFBP-1 phosphorylation where IGFBP-1 sequesters IGF-1 and reduces IGF-1R signaling, to inhibit cellular growth and proliferation. AMPK (AMP-activated kinase), TSC2 (tuberous sclerosis complex 2), insulin-like growth factor (IGF) binding protein 1 (IGFBP-1), mechanistic target of rapamycin (mTOR), eukaryotic translation initiation factor 4E-binding protein 1 (4E-BP1), ribosomal protein S6 790 kinase 1 (P70S6K1). Created with BioRender.com.

Based on our findings, we suggest that fetal hypoglycemia decreases fetal growth by the activation of AMPK and TSC2, which in turn inhibit mTORC1 signaling. This results in IGFBP-1 hyperphosphorylation and decreased IGF-1 bioavailability leading to impaired fetal growth (Fig. 10). A previous report linking AMPK to increased secretion of IGFBP-1 (Lewitt 2001) provides general support of this model.

In conclusion, we report that IGFBP-1 hyperphosphorylation induced by glucose deprivation is mediated via the AMPK–mTORC1 pathway. These findings provide evidence for a novel mechanistic link between limitation in glucose availability and impaired fetal growth via increased levels of IGFBP-1 phosphorylation and secretion. This information may help us better understand the pathophysiology of FGR and develop potential interventions to counter impaired fetal growth and its short- and long-term adverse outcomes.

## Supplementary materials

Supplemental Figure 1

Supplemental Figure 2

Supplemental Figure 3

Supplemental Figure 4

## Declaration of interest

The authors report no conflicts of interest related to this work.

## Funding

This work did not receive any specific grant from any funding agency in the public, commercial, or not-for-profit sector.

## Author contribution statement

JK, MUK, IH, and VH contributed to the conception and design of the work. JK, MUK, IH, and VH contributed to the acquisition, analysis and interpretation of data for the work. JK, MUK, IH, and VH contributed to the drafting the work and revising it critically for important intellectual content. All authors approved the final version of the manuscript and agree to be accountable for all aspects of the work in ensuring that questions related to the accuracy or integrity of any part of the work are appropriately investigated and resolved; and all persons designated as authors qualify for authorship, and all those who qualify for authorship are listed.
