# Supplementary figures and images for: AMPK–mTORC1 pathway mediates hepatic IGFBP-1 phosphorylation in glucose deprivation: a potential molecular mechanism of hypoglycemia-induced impaired fetal growth
Source: J Mol Endocrinol. 2024 Jan 31;72(3):e230137. doi: 10.1530/JME-23-0137 (PMC10895286; doi:10.1530/JME-23-0137)

# Supplemental Figure 1

**A**

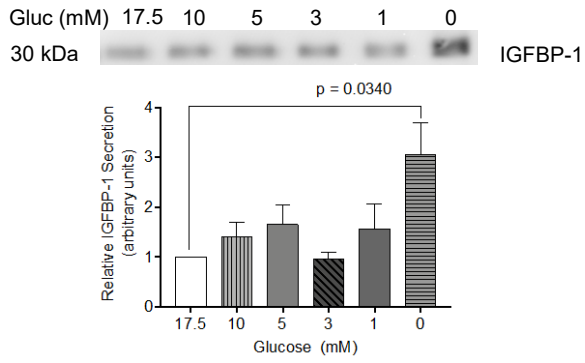

**B**

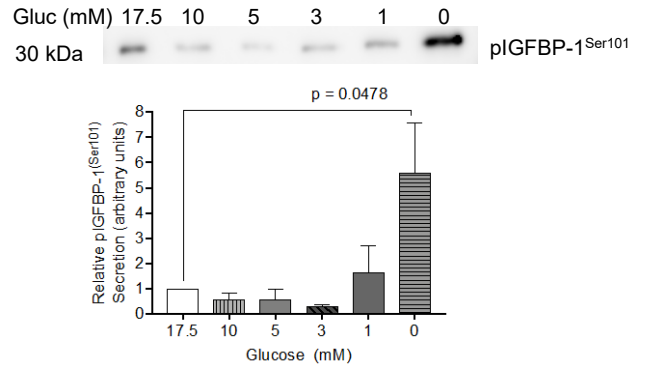

**C**

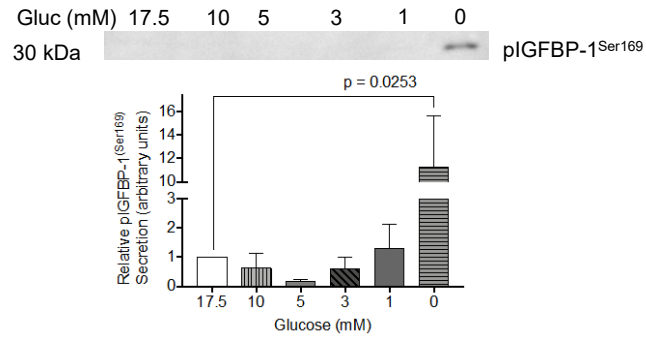

Supplement: Supplemental Figure 1 [file supplementary_figure_1.pdf]

**Supplemental Figure 2**

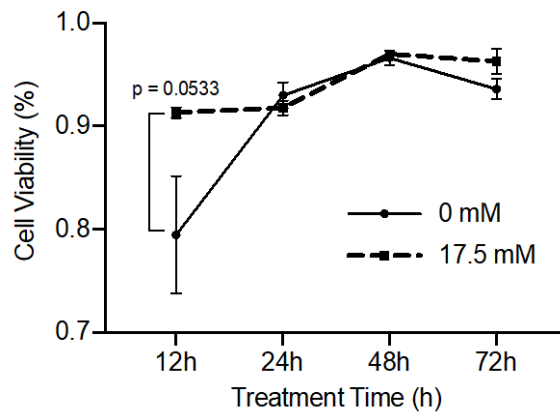

Supplement: Supplemental Figure 2 [file supplementary_figure_2.pdf]

# Supplemental Figure 3

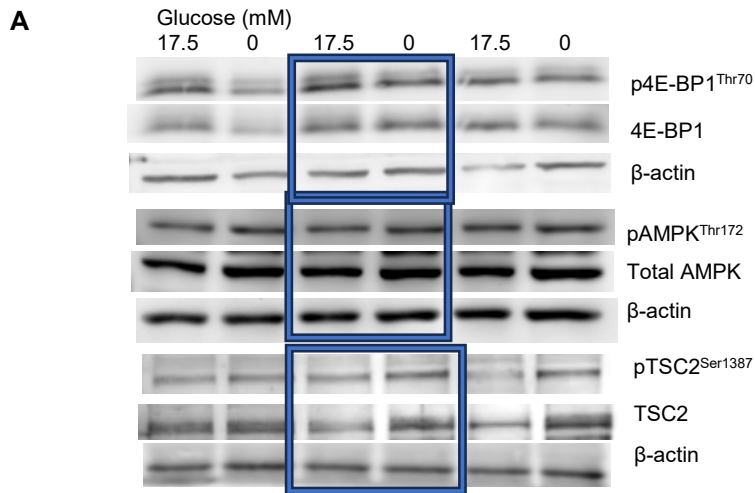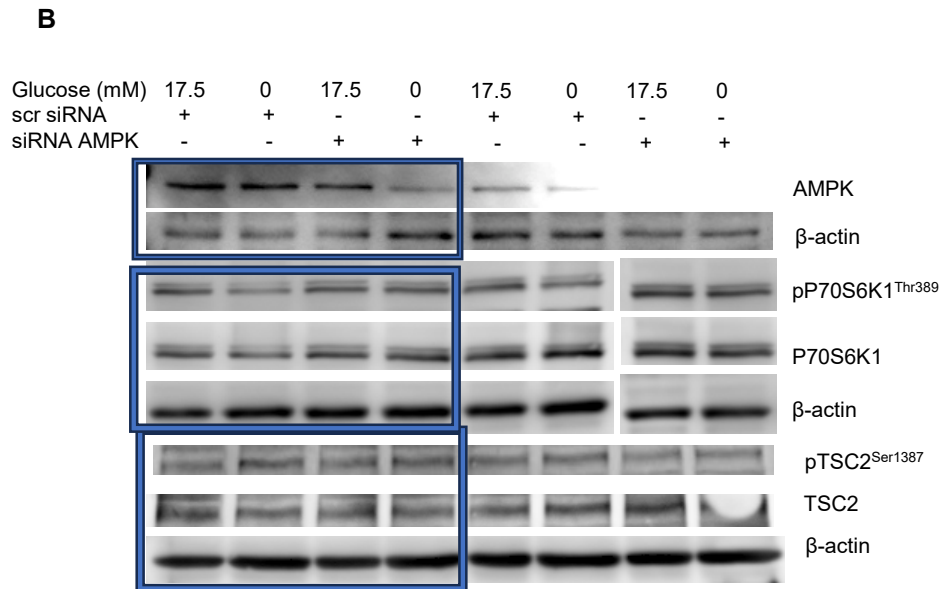

Supplement: Supplemental Figure 3 [file supplementary_figure_3.pdf]

Supplemental Figure 4

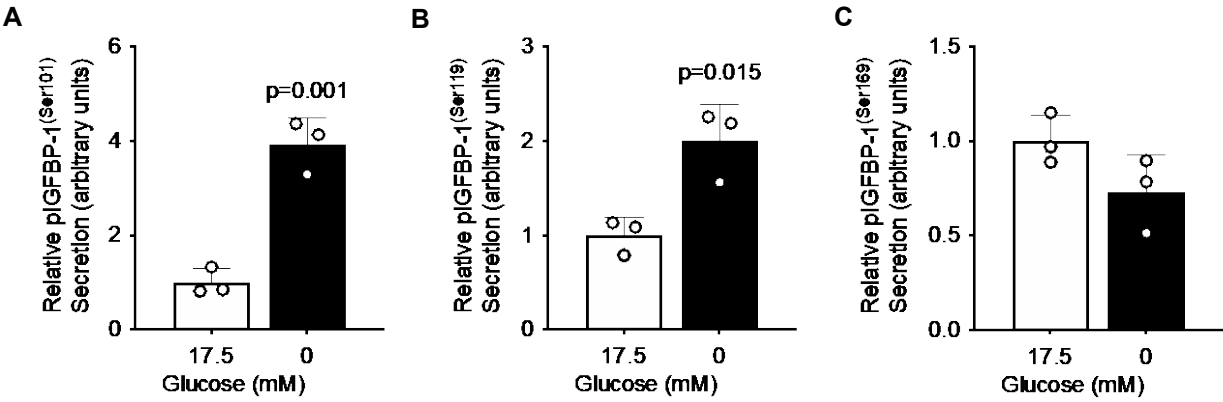

Supplement: Supplemental Figure 4 [file supplementary_figure_4.pdf]
